# Artemisinin and Its Derivate Alleviate Pulmonary Hypertension and Vasoconstriction in Rodent Models

**DOI:** 10.1155/2022/2782429

**Published:** 2022-06-17

**Authors:** Changlei Bao, Qian He, Hui Wang, Yanan Sun, Yahang Xu, Yan Pan, Yadan Hu, Shichuang Zheng, Shuxin Liang, Ang Luo, Tanzilan Nahar, Jiwang Chen, Haiyang Tang, Ying Han

**Affiliations:** ^1^College of Veterinary Medicine, Northwest A&F University, Yangling, Shaanxi, China; ^2^State Key Laboratory of Respiratory Disease, Guangdong Key Laboratory of Vascular Disease, Guangzhou Institute of Respiratory Health, The First Affiliated Hospital of Guangzhou Medical University, Guangzhou, China; ^3^Key Laboratory of Targeted Intervention of Cardiovascular Disease, Collaborative Innovation Center for Cardiovascular Disease Translational Medicine, and Department of Physiology, Nanjing Medical University, Nanjing, China; ^4^Center for Cardiovascular Research, University of Illinois at Chicago, IL 60612, USA; ^5^Department of Medicine, University of Illinois at Chicago, IL 60612, USA

## Abstract

**Background:**

Pulmonary arterial hypertension (PAH) is a complex pulmonary vasculature disease characterized by progressive obliteration of small pulmonary arteries and persistent increase in pulmonary vascular resistance, resulting in right heart failure and death if left untreated. Artemisinin (ARS) and its derivatives, which are common antimalarial drugs, have been found to possess a broad range of biological effects. Here, we sought to determine the therapeutic benefit and mechanism of ARS and its derivatives treatment in experimental pulmonary hypertension (PH) models.

**Methods:**

Isolated perfused/ventilated lung and isometric tension measurements in arteries were performed to test pulmonary vasoconstriction and relaxation. Monocrotaline (MCT) and hypoxia+Su5416 (SuHx) were administered to rats to induce severe PH. Evaluation methods of ARS treatment and its derivatives in animal models include echocardiography, hemodynamics measurement, and histological staining. *In vitro*, the effect of these drugs on proliferation, viability, and hypoxia-inducible factor 1*α* (HIF1*α*) was examined in human pulmonary arterial smooth muscle cells (hPASMCs).

**Results:**

ARS treatment attenuated pulmonary vasoconstriction induced by high K^+^ solution or alveolar hypoxia, decreased pulmonary artery (PA) basal vascular tension, improved acetylcholine- (ACh-) induced endothelial-dependent relaxation, increased endothelial nitric oxide (NO) synthase (eNOS) activity and NO levels, and decreased levels of NAD(P)H oxidase subunits (NOX2 and NOX4) expression, NAD(P)H oxidase activity, and reactive oxygen species (ROS) levels of pulmonary arteries (PAs) in MCT-PH rats. NOS inhibitor, L-NAME, abrogated the effects of ARS on PA constriction and relaxation. Furthermore, chronic application of both ARS and its derivative dihydroartemisinin (DHA) attenuated right ventricular systolic pressure (RVSP), Fulton index (right ventricular hypertrophy), and vascular remodeling of PAs in the two rat PH models. In addition, DHA inhibited proliferation and migration of hypoxia-induced PASMCs.

**Conclusions:**

In conclusion, these results indicate that treatment with ARS or DHA can inhibit PA vasoconstriction, PASMC proliferation and migration, and vascular remodeling, as well as improve PA endothelium-dependent relaxation, and eventually attenuate the development and progression of PH. These effects might be achieved by decreasing NAD(P)H oxidase generated ROS production and increasing eNOS activation to release NO in PAs. ARS and its derivatives might have the potential to be novel drugs for the treatment of PH.

## 1. Introduction

Pulmonary arterial hypertension (PAH) is a progressive and life-threatening cardiopulmonary vascular disease characterized by elevated pulmonary vascular resistance, increased pulmonary vascular pressure, and irreversible right heart failure that ultimately leads to premature death [1]. The major causes of elevated pulmonary vascular resistance include endothelial dysfunction, medial layer hypertrophy, increased myogenic tone, and pulmonary artery wall stiffness, as well as sustained pulmonary vasoconstriction [2, 3]. Recently, pharmacological agents targeting pulmonary artery vasoconstriction through vasodilators have been developed and applied in the clinic including prostanoids (epoprostenol or iloprost), endothelin receptor blockers (bosentan or sitaxentan), and/or phosphodiesterase-5 inhibitors (sildenafil) and soluble guanylate cyclase stimulators (riociguat) [4–6]. Despite the benefits of these drugs in the management of pulmonary hypertension (PH), their high cost, adverse side effects, and inconvenient drug administration routes are a major hindrance in receiving proper medical intervention. For example, patients receiving epoprostenol with a central venous catheter have an increased risk of infections and the potential for the severe complication of sepsis [7, 8]. In addition, the average cost for tadalafil, the commonly used PAH medication in Canada, is around Can$881. However, the cost for ambrisentan oral tablet is around Can$4,028 per month in Canada [7]. In China, 38% of PAH patients paid over 5000 yuan per month for similar treatments. Due to the financial burden, over 50% of patients did not receive any type of PAH-specific therapies [9]. Therefore, it is urgent to develop a novel therapeutic drug that is safer, affordable, and therapeutically more effective for patients with PAH.

Artemisinin (ARS) and its derivative, dihydroartemisinin (DHA), have been found to be useful to prevent the experimental PH [10, 11]. ARS and DHA are widely used as antimalarial drugs with relatively low toxicity. ARS is extracted from a traditional Chinese herb called Artemisia annua, and DHA is a known artemisinin derivative. It has been reported that NAD(P)H oxidase-reactive oxygen species (ROS) and endothelial nitric oxide (NO) synthase- (eNOS-) NO signaling pathways are involved in the enhanced pulmonary vasoconstriction and endothelial dysfunction in PH [12–15]. Previous study has shown that DHA prevents monocrotaline- (MCT-) mediated pulmonary vascular remodeling in rats via inhibiting PASMC proliferation and migration [10]. ARS or its derivatives mitigate ROS levels [16] and enhance the phosphorylation of eNOS [17]. However, it is unknown whether ARS can attenuate pulmonary vasoconstriction and improve endothelial function at the tissue level through NAD(P)H oxidase-ROS or eNOS-NO signaling pathways in pulmonary hypertension. Furthermore, it is also undetermined whether ARS or DHA can reverse experimental PH in animal models. In addition, the therapeutic benefit of ARS and DHA on PAH has not been examined in hypoxia+Su5416- (SuHx-) mediated severe PH rat model which is commonly used to study PH as it can resemble the pathology of human PAH [18, 19].

To address these questions, we employed a well-established mouse-isolated lung perfusion model to examine the effects of ARS on acute hypoxia or high K^+^-mediated pulmonary vasoconstriction. We also employed rat models of severe PH mediated by MCT or SuHx to examine if ARS or DHA can attenuate PH. In addition, we studied whether ARS improves endothelium-dependent relaxation and decreases NAD(P)H oxidase activity and superoxide anions levels, as well as increases eNOS activity and NO production of MCT-mediated PH in rats.

## 2. Materials and Methods

### 2.1. Ethics Statement

The animal care and experimental procedures were approved by the Institutional Animal Care and Use Committee of Northwest Agriculture and Forestry University (Yangling, China), Guangzhou Medical University (Guangzhou, China), and Nanjing Medical University (Nanjing, China). All animals were bred and housed in the animal research center of the university. Standardized protocols were followed for rodent experiments. All the surgical procedures were done under full anesthesia. At the end of each study, animals were euthanized by the removal of the hearts and lungs with 2-5% isoflurane anesthesia.

### 2.2. ARS or DHA Treatment in MCT-Induced Pulmonary Hypertension Rat Model

Male rats (200 ± 20 g) were subcutaneously injected with MCT (MedChemExpress) at a 50 mg/kg single dose to induce PH as previously described [20–22], and the control rats were injected with equivalent amounts of saline. For ARS treatment, rats were randomly divided into 4 groups as follows: (1) control (Cont)+DMSO group; (2) Cont+ARS group; (3) MCT+DMSO group; and (4) MCT+ARS group. Following the administration of MCT or saline, ARS (Meilunbio) was given to the rats by intraperitoneal administration at 60 mg/kg/day for 28 days, and DMSO was the control vehicle for ARS. Similar to the ARS treatment, for DHA treatment, the rats were also randomly divided into 4 groups as follows: (1) Cont+DMSO group; (2) Cont+DHA group; (3) MCT+DMSO group; and (4) MCT+DHA group. Following the administration of MCT or saline, DHA (Tokyo Chemical Industry) (14 mg/kg/day) or DMSO was given to the rats by intraperitoneal administration for 28 days.

### 2.3. ARS Treatment in SuHx-Induced Pulmonary Hypertension Rat Model

Male rats (200 ± 20 g) were randomly divided into 4 groups: (1) normoxia vehicle+DMSO group (DMSO); (2) Su5416 combined with hypoxia-exposed group (SuHx); (3) SuHx+normoxia+ARS group (SuHx-ARS1); and (4) SuHx+ARS+normoxia group (SuHx-ARS2). To build a SuHx-PH model, the rats were injected with 20 mg/kg of Su5416 (MedChemExpress) subcutaneously and exposed to chronic normobaric hypoxia at 10% O_2_ inside a chamber for 3 weeks as previously described [23], then followed by renormoxia (21% O_2_) for 6 additional weeks. The application of ARS (60 mg/kg/day) in the SuHx-ARS1 group was started from the 7^th^ week after Su5416 injection and lasted for 3 weeks under normoxia condition. The rats in the SuHx-ARS2 group were treated with ARS under normoxia condition immediately after hypoxia exposure for 3 weeks and then situated in normoxia condition for 3 additional weeks. The rats in the normoxia control group were subjected to equal volume of DMSO and exposed in normoxia identical conditions for 9 weeks. All animals were sacrificed at the end of the 9^th^ week.

### 2.4. Measurement of Pulmonary Arterial Pressure (PAP) in Isolated Perfused/Ventilated Mouse Lung

The PAP was measured using the isolated perfused/ventilated mouse lung system (Harvard Apparatus) as described previously [24–26]. In brief, C57BL/6 mice (20–25 g) were anesthetized with sodium pentobarbital solution (50 mg/kg) via intraperitonelly injection and then were ventilated via ventilatory control module- (VCM-) R with timer counter module (TCM) after tracheostomy. The parameter settings were as follows: respiratory rate (80 breaths/min), tidal volume (10 mL/kg), and positive end-expiratory pressure (2 mm H_2_O). Heparin solution (20 IU) was slowly injected to the right ventricle to prevent blood clots in the lung after median sternotomy. The pulmonary circulation was monitored in a closed pipeline via a peristaltic pump after cannulating in the main pulmonary artery and the left atrium. PAP was measured via a pressure transducer P75 (Type 379) which was attached to the PA catheter. The lung vasculature was perfused with 37°C Krebs-Ringer solution (pH 7.4) with the following composition (mM): KCl 4.3, NaCl 120, KH_2_PO_4_ 1.1, NaHCO_3_ 19, MgCl_2_ 1.2, CaCl_2_ 1.8, glucose 10, and 20% (*v*/*v*) fetal bovine serum. Pulmonary vasoconstriction was induced by 40 mmol/L K^+^ solution (NaCl was replaced by an equimolar amount of KCl) or hypoxic mixture (1% O_2_, 5% CO_2_, and equilibrium N_2_). Pulmodyn (V2.0) was used for data acquisition and storage. The experiments were performed after the basic PAP had stabilized for 30-60 min.

### 2.5. Isometric Tension Measurements in Arteries

Isometric tension experiment was performed to evaluate vascular function as we previously reported [27–29]. Briefly, the third-order PA from the rats were isolated and cut into 2 mm segments in Krebs-Henseleit solution (the constituents of it can be found in our previous study [27, 28]). Then, we mounted the arterial rings (1 arterial ring/artery/rat was used) in a four-chambered myograph (620 M, DMT, Denmark) and set a resting tension at 0.1 g. After equilibration, the 6 doses of acetylcholine (ACh) (10^−9^~10^−4^ M) was administered to evaluate arterial relaxation after prostaglandin F2*α*- (PGF 2*α*-) induced arterial ring contraction (1-5 *μ*M). The degree of relaxation is shown as a percentage of PGF 2*α*-induced contraction. The pretreatment of DMSO or ARS (the final concentration in chamber is 20 *μ*M) was added, respectively, into myograph chamber 1 h before PGF 2*α*-induced contraction.

### 2.6. Measurement of eNOS Activity and NO Level of Arteries

The third-order PA samples with the same length were isolated from rats and incubated in Krebs-Henseleit solution contained with 0.1% DMSO and ARS (20 *μ*M) for 1 h. Afterwards, the activity of eNOS was evaluated by the ability of converting L-arginine to NO using a Nitric Oxide Synthase Assay Kit (Beyotime) following the manufacturer's instruction. The second way to evaluate the eNOS activity was done by measuring p-eNOS/T-eNOS.

For NO level measurement, after incubation of drugs for 1 h, the PA tissues were flash-frozen in liquid nitrogen and stored at -70°C. After that, the artery tissues were homogenized in RIPA lysis buffer and centrifuged. Next, the total protein in the homogenate supernatant was extracted and measured using a protein assay kit (Beyotime). The NO production in PA tissues homogenate supernatant was evaluated by a Nitrate/Nitrite Colorimetric Assay Kit (Cayman) following the manufacturer's instruction. During all operation procedures, the samples were kept in the ice to prevent oxidisation.

### 2.7. Measurement of NAD(P)H Oxidase Activity and Superoxide Anion Levels of Arteries

After PA incubation with DMSO or ARS for 1 h, the NAD(P)H oxidase activity and superoxide anions levels of PA were measured by enhanced lucigenin-derived chemiluminescence method, as described in our previous reports [30]. Briefly, the light emissions produced by the reactions between lucigenin (5 *μ*M) and the superoxide anions in PA homogenate supernatant were first detected by a luminometer (20/20n, Turner, CA, USA) once every minute for 10 minutes in order to measure superoxide anions level. The detected luminescence value after SOD was added into PA homogenate supernatant to remove the existing superoxide anions that was set as blank. Next, to measure NAD(P)H oxidase activity, NAD(P)H (100 *μ*M) was added first into the medium as a substrate for it to react with NAD(P)H oxidase so it can generate new superoxide anions, which would then be quantified and detected by the luminometer. The difference values between it and the previous value of superoxide anions level were used to indicate the activity of NAD(P)H oxidase. The values represented NAD(P)H oxidase activity and superoxide anions levels were expressed as a mean light unit (MLU) per minute per milligram of protein.

### 2.8. Transthoracic Echocardiography

Transthoracic echocardiography was used to measure right ventricular (RV) function via the Vevo 2100 high-resolution imaging system (Visual Sonics Inc.) equipped with a cardiovascular scan transducer (MS-250, 13-24 MHz) as previously described [31]. Briefly, the rats were maintained with 1-2% anesthesia via a facemask and warmed with a heated pad to keep normal continuous heart and respiratory rates. Right ventricle free wall thickness (RVFWT) that correlates with right ventricular hypertrophy (RVH) was measured at the end of a diastole in the right parasternal short-axis (PSAX) mitral valve level using two-dimensional (2D) M-mode. To determine RV systolic function, RV fractional area change (RVFAC) and tricuspid annular plane systolic excursion (TAPSE) were measured. RVFAC obtained from the apical four-chamber view using B-mode was calculated by the equation: RVFAC (%) = RV end diastolic chamber area (RVEDA) − RV end systolic chamber area (RVESA)/RVEDA. In the apical four-chamber view, TAPSE was measured by the amount of longitudinal excursion of the annulus from end-diastole to end-systole under M-mode. Pulse-wave Doppler echo was used to record the pulmonary blood outflow tract at the level of the aortic valve in the parasternal short-axis view to measure pulmonary acceleration time (PAT) and pulmonary ejection time (PET).

### 2.9. Hemodynamic Evaluation

Following echocardiography, hemodynamic evaluations, including RV pressure and Fulton index measurement, were performed as previously described [3, 32]. During operation, the rats were maintained with 1-2% anesthesia via a facemask and kept warm with a heated pad. RV catheterization was performed by inserting a catheter containing a flexible Millar pressure transducer (SPR-513) via right jugular vein into the RV for real-time monitoring of right ventricular pressure (RVP). During the measurement, the rats maintained a normal continuous heart rate and respiratory rate. RVP, RVSP, RV±dP/dt, and heart rate were collected and analyzed using the PowerLab data acquisition system (AD Instruments) and LabChart 8.0 software. The mean pulmonary arterial pressure (mPAP) was estimated according to the equation mPAP = 0.61 RVSP + 2 mmHg [33]. After the animals were euthanized, the lungs were flushed from the right ventricle with chilled PBS. Fulton index was indicated as the ratio of RV weight to the left ventricle (LV) plus septum (S) weight [(RV/LV + S)] and to the body weight (BW) [(RV/BW)].

### 2.10. Histopathology Assessment

To determine the histological modifications of pulmonary vascular remodeling or RV hypertrophy, the RV and lungs were immediately immersed in 4% paraformaldehyde, embedded in paraffin, sectioned at 4 *μ*m thickness, and then mounted onto slides according to the common procedures protocol. Subsequently, all the slides were stained by hematoxylin and eosin (H&E) to determine cardiomyocyte hypertrophy and pulmonary artery media thickness. Meanwhile, the myocardial sections were also stained with Masson's trichrome staining which indicated the scales of collagen deposition fibrosis using standard histochemistry procedures according to the manufacturers' instruction. The medial wall thickness of pulmonary arterioles (diameter 50-100 *μ*m) expressed as a ratio of wall area/total vessel area was measured in H&E images of lung cross-sections according to the previous methods [34]. RV interstitial fibrosis ratio (%area) was calculated as (blue collagen area/tissue area). A random selection of five fields of each section was calculated for analysis per group. All the results were assessed by ImageJ software (National Institutes of Health). All of the analyses were performed in a blinded manner.

### 2.11. Immunohistofluorescence Staining

Lung tissue sections were dewaxed, rehydrated, and then washed with PBS. After antigen retrieval, the sections were stained for anti-*α*-smooth muscle actin (*α*-SMA, Abcam, #ab7817) antibody and antiproliferating cell nuclear antigen (PCNA, Cell Signaling Technology, #2586) antibody or anti-*α*-SMA antibody and antiplatelet endothelial cell adhesion molecule-1 antibody (PECAM-1/CD31, Abcam, #ab182981) with appropriate fluorescent secondary antibodies and DAPI mounting medium. All slides were scanned using an AperioScanScope (Leica), and representative images were taken at ×100 by an individual blinded to experimental condition.

### 2.12. Cell Culture

Human pulmonary arterial smooth muscle cells (hPASMCs) were purchased from Lonza (Walkersville, MD, USA). HPASMCs were cultured in SmGM-2 medium (Lonza) supplemented with SmGM-2 SingleQuots (Lonza) and antibiotics (100 IU/mL penicillin and 100 *μ*g/mL streptomycin) according to the manufacturer's instruction. The cells at passages 5–8 were used for the experiments. For the normoxic group, the cells were maintained in a humidified atmosphere of 5% CO_2_ and 95% air at 37°C, while the cells from the hypoxic group were subjected to 1.5% O_2_ at 37°C for 48–72 h.

### 2.13. Cell Viability Assay

Cell viability was determined by Cell-Counting Kit-8 (Biotime, #C0043) assay according to the manufacturer's protocol. Briefly, the cells (5 × 10^3^/well) were seeded in 96-well culture plates and cultured overnight. The cells were treated with different concentrations of DHA (0, 5, 10, 20, 40, 80, and 160 *μ*M) for 24, 48, and 72 h, respectively. CCK-8 solution (10 *μ*L) was added to each well of the plate and then incubated for 2 h in the incubator for measurement of absorbance at 450 nm using a microplate reader.

### 2.14. Western Blotting

The cells were harvested, washed twice with cold PBS, and then lysed in RIPA buffer (Nanjing KeyGen Biotech) with a protease and phosphatase inhibitor cocktail (Roche) and centrifuged at 12,000 rpm for 15 min at 4°C. Protein concentration in the lysates was determined by the bicinchoninic acid (BCA) Protein Assay Kit (Nanjing KeyGen Biotech) using the manufacturer's recommendations. Equal amounts of protein (30 *μ*g) from each sample was resolved by SDS-PAGE (12% polyacrylamide gel) and subsequently transferred onto a 0.22 *μ*m polyvinylidene difluoride (PVDF) membrane (Millipore), which was blocked with 5% nonfat milk or BSA in TBST buffer and incubated with primary antibodies overnight at 4°C. The primary antibodies were as follows: anti-NOX2 (Abcam, #ab129068), anti-NOX4 (Abcam, #ab133303), anti-eNOS (Invitrogen, PA1-037), anti-phospho-eNOS (Ser1177) (Invitrogen, #PA5-17917), anti-HIF1*α* (Abcam, #ab179483), anti-PCNA (Cell Signaling Technology, #2586), anti-Ki67(Abcam, #ab92742), anti-*β*-actin (Proteintech, #66009), and anti-*β*-tubulin (Abcam, #ab6046). Then, the membranes were washed three times with TBST for 10 min each, followed by incubation in anti-rabbit or anti-mouse conjugated to horseradish peroxidase-IgG secondary antibody for 1 h at room temperature. Finally, the blot was detected by the Western Super Signal chemiluminescent substrate (Bio-Rad) and automatic chemiluminescence image analysis system (Tanon 4800). The densitometry analysis was performed using ImageJ software. Band intensity was normalized to *β*-actin or *β*-tubulin controls and is expressed in arbitrary units.

### 2.15. Flow Cytometric Assay

HPASMC proliferation was assessed with a Cell-Light 5-ethynyl-2′-deoxyuridine (EdU) Apollo488 in Vitro Flow Cytometry Kit (Ribobio, #C10338-3). Cell suspension was grown on 6-well plates at a density of 5 × 10^4^ cells/well. After overnight attachment and serum-free starvation, the cells were treated with DMSO or DHA before exposure to hypoxia (1.5% O_2_) for 48 h. During the final 6 h of incubation, 10 *μ*M EdU was added to the cell culture media. Detection of EdU incorporated into newly synthesized DNA was performed according to manufacturer's instruction.

### 2.16. Cell Migration

To determine the migration ability of hPASMCs, the 2 Well Culture-Inserts (IBIDI, #80209) were transferred to new 35 mm culture dishes to perform a transwell migration assay. The cells were prepared as suspension, adjusted at a cell concentration at 3 × 10^5^ cells/mL, and then applied 70 *μ*L into each well to seed the cells. When it formed an optically confluent monolayer within 24 h, the Culture-Insert 2 Well was gently removed with sterile tweezers, and the dishes were filled with cell-free medium DMSO or DHA (20 *μ*M). After drug treatment, a cell-free gap is created in which the cell migration can be visualized. Photos were taken every two hours until 12 h to observe the migration distance between normal and DHA treatment groups. The migration area analysis was assessed using ImageJ software.

### 2.17. Data and Statistical Analysis

The numbers in figure legends indicate biological replicates performed in each experiment. The data were expressed as mean ± SEM. Comparison of means between two or more groups was achieved by the unpaired, two-tailed Student's *t*-test or one-way ANOVA by using GraphPad Prism version 8. *P* < 0.05 was considered to indicate a statistically significant difference.

## 3. Results

### 3.1. ARS Treatment Attenuated High K^+^ or Alveolar Hypoxia-Induced Pulmonary Vasoconstriction

**Figure 1:**
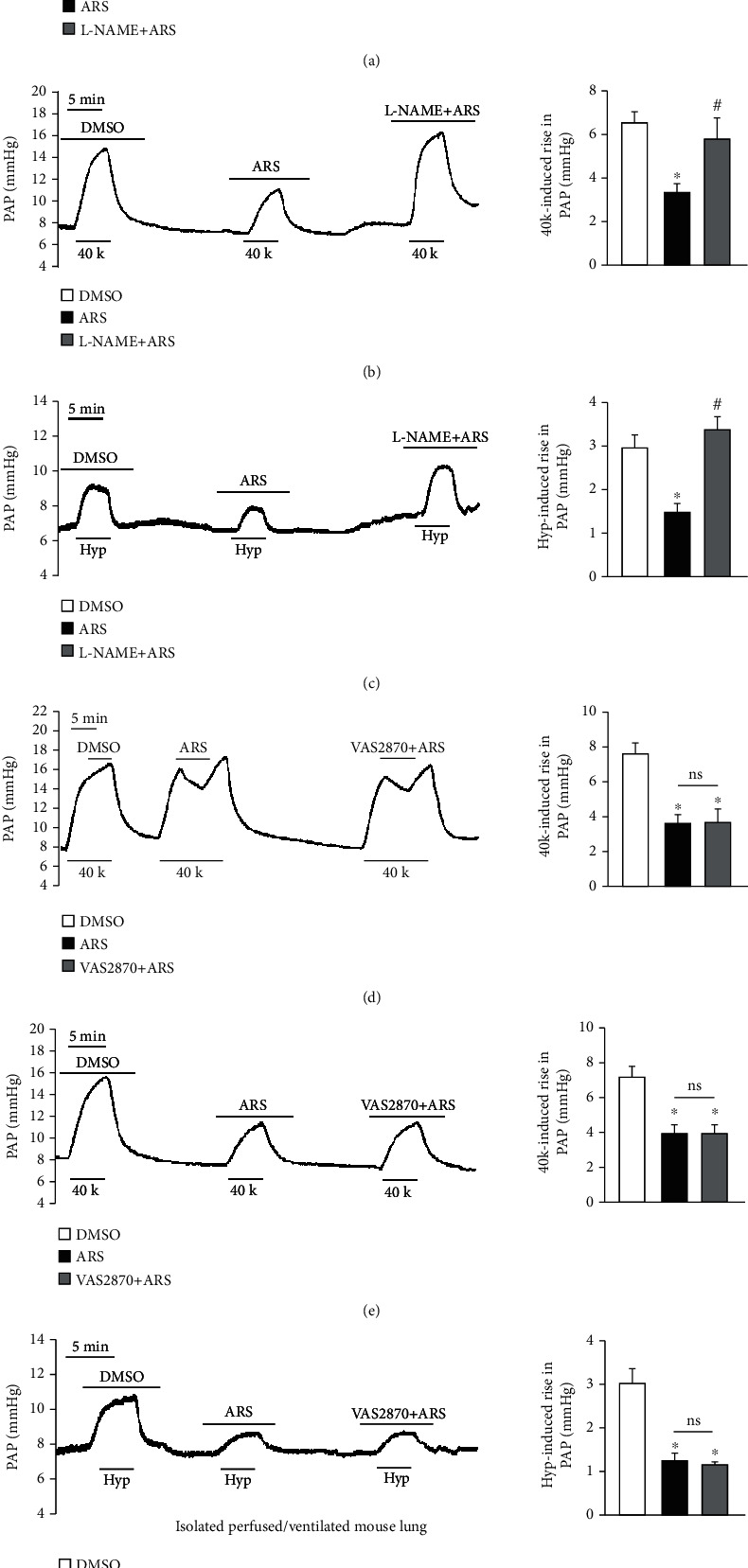
ARS attenuates high K^+^ solution and hypoxia-induced pulmonary vasoconstriction. Representative records (left panel) and summarized data (right panel) of pulmonary arterial pressure (PAP) in isolated perfused/ventilated lung preparation showing the relaxing effects of DMSO, ARS (200 *μ*M), and L-NAME (10 *μ*M)+ARS (200 *μ*M) on the contraction induced by high K^+^ solution (40 mM) (40K) (a); the effect of DMSO, ARS (200 *μ*M), and L-NAME (10 *μ*M)+ARS (200 *μ*M) pretreatment on the contraction induced by 40K (b) and the contraction induced by hypoxia (Hyp) (c); the relaxing effects of DMSO, ARS (200 *μ*M), and VAS2070 (10 *μ*M)+ARS (200 *μ*M) on the contraction induced by 40K (d); the effect of DMSO, ARS (200 *μ*M), and VAS2070 (10 *μ*M)+ARS (200 *μ*M) pretreatment on the contraction induced by 40K (e) and the contraction induced by Hyp (f). Values are expressed as mean ± SEM. ^∗^*P* < 0.05, compared with DMSO. ^#^*P* < 0.05, compared with ARS. Ns: no significant difference. *n* = 3 − 6 for each group.

To investigate whether ARS can directly affect PAP, we examined the effects of ARS on high K^+^ solution or hypoxia-induced pulmonary vasoconstriction using isolated perfused/ventilated mouse lung. Firstly, we confirmed that PAP significantly increased under 40 mM K^+^ solution (40K) for 5 min. When the 40K-mediated increase in PAP reached a plateau, compared to DMSO, ARS (200 *μ*M) application for 5 min induced a significant drop of PAP, suggesting that ARS has the ability to relax PA (Figures 1(a) and 1(d)). Pretreatment of the isolated lung by continuous perfusion of ARS for 10 min also significantly attenuated the 40K-induced increase in PAP (Figures 1(b) and 1(e)). Next, we examined the effect of ARS on acute hypoxia-induced pulmonary vasoconstriction. In DMSO control group, 1% O_2_ acute alveolar hypoxia (Hyp) for 5 min significantly increased the PAP in isolated perfused/ventilated lung, which returned to baseline under normoxic conditions. In contrast, pretreatment with ARS for 10 min significantly attenuated the ability of hypoxia to increase PAP (Figures 1(c) and 1(f)). These data demonstrated that ARS treatment attenuated high K^+^ or alveolar hypoxia-induced pulmonary vasoconstriction. To further explore the mechanism of ARS action, pretreatment with L-NAME (eNOS inhibitor) or VAS2870 (NAD(P)H oxidase inhibitor) before ARS was performed. We found that L-NAME pretreatment significantly inhibited the effects of ARS on PAP in both 40K- and Hyp-induced pulmonary vasoconstriction (Figures 1(a)–1(c)). However, the pretreatment with VAS2870 had no significant influence on the effects of ARS (Figures 1(d)–1(f)). These results suggest that ARS administration attenuates vascular constriction in a NO-dependent manner.

### 3.2. ARS Treatment Attenuated the Development of MCT-Induced Pulmonary Hypertension

**Figure 2:**
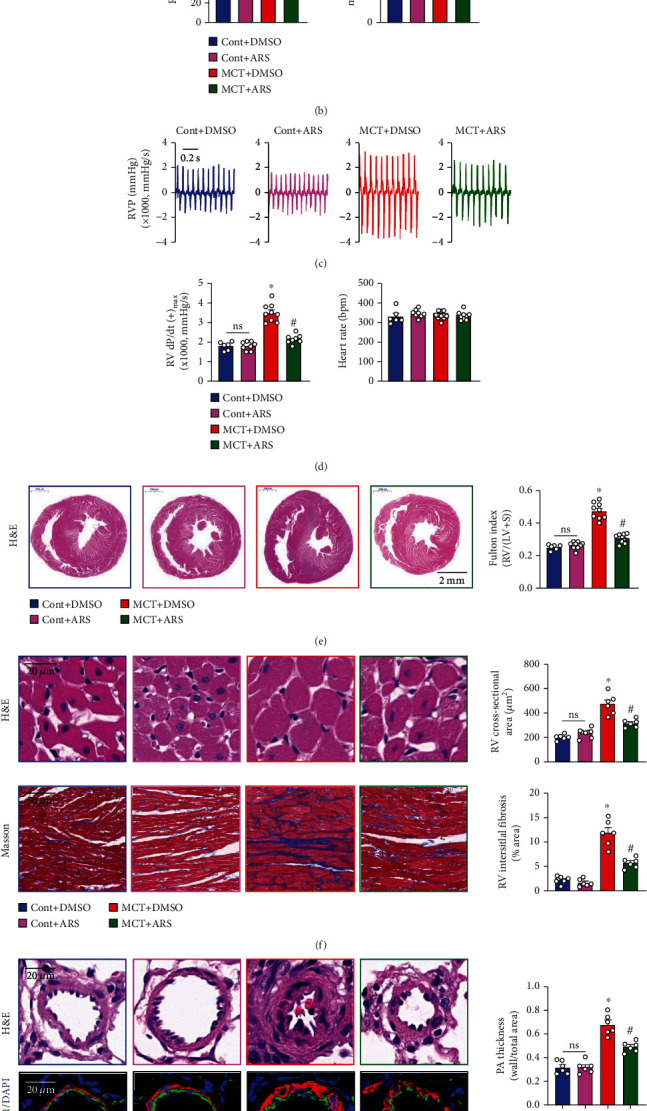
ARS treatment improves the development of MCT-induced pulmonary hypertension in rats. Male rats (200 ± 20 g) were subcutaneously injected with MCT at a 50 mg/kg single dose and were simultaneously administrated with ARS (60 mg/kg/day, i.p.) for 4 weeks. (a) Representative tracings showing RVP. (b) Summarized data showing RVSP and mPAP. (c) Representative tracings showing RV ± dP/dt. (d) Summarized values of RV dp/dt(+)_max_ and heart rate in Cont and MCT-treated rats receiving DMSO or ARS. (e) Representative H&E images showing right ventricular wall hypertrophy and right ventricular cavity dilation (left panel), summarized data showing Fulton index [RV/(LV + S)] (right panel). (f) Representative H&E staining images of myocardial fiber cross-section (left top panel, scale bar = 20 *μ*m) and Masson-stained images (left bottom panel, scale bar = 50 *μ*m) showing rat myocardial cell morphology and collagen fibers (blue indicates collagen deposition and red represents muscle fibers), respectively, summarized data showing RV cross-sectional area (right top panel) and RV interstitial fibrosis ratio (right bottom panel, blue collagen area/tissue area). (g) Representative H&E images and immunofluorescence images (*α*-SMA, marker of smooth muscle layer, was shown in red; CD31, marker of endothelial muscle layer shown in green, nucleus shown in green) of the cross-section of small PA in the lung sections of Cont and MCT-PH rats receiving DMSO or ARS (left panel). Scale bar, 20 *μ*m. Bar graph showing media thickness of the small PAs (right panel). Values are mean ± SEM. ^∗^*P* < 0.05, compared with Cont+DMSO. ^#^*P* < 0.05, compared with MCT+DMSO. Ns: no significance. The numbers of experiments (*n*) for each group are indicated in each bar.

Persistent elevated pulmonary arterial pressure leads to elevated right ventricular systolic pressure (RVSP), cardiomyocyte hypertrophy, and fibrosis in PH [34]. Firstly, the effect of ARS *in vivo* experiment was further performed on MCT-induced PH rat models. Compared with Cont+DMSO group, MCT+DMSO treatment induced a significant increase in RVSP, mean pulmonary arterial pressure (mPAP, estimated according to the equation: mPAP = 0.61 RVSP + 2 mmHg) [33], and RV dP/dt(+)_max_ (evaluation for RV contractility) (Figures 2(a)–2(d)) but had no significant influence on the heart rates (Figure 2(d)). Increased RVSP/mPAP was associated with significant Fulton index showed by H&E staining ventricular images and weight ratio of RV/(LV + S) (Figure 2(e)) in rats. Notably, treatment with ARS decreased the RVSP (Figure 2(b)) and right ventricular hypertrophy (Figure 2(e)) of MCT-PH rats. From the H&E staining results in myocardial cell cross-section, we further confirmed that ARS significantly inhibited cardiomyocyte hypertrophy of the right ventricle (Figure 2(f)). Additionally, the Masson staining of the right ventricular and semiquantitative analysis of myocardial fibrosis showed that the MCT treatment induced the deposition of blue collagen fibers (Figure 2(f)), suggesting severe perivascular and myocardium fibrosis. ARS treatment was also markedly declined RV interstitial fibrosis (Figure 2(f)). H&E staining and immunofluorescence staining with CD31&*α*-SMA antibodies in PAs have shown that compared with Cont+DMSO group, PA media in MCT-PH rats significantly proliferated and became thicker, while the lumen diameter of PA was reduced, resulting in stenosis or even complete occlusion, indicating the occurrence of vascular remodeling. ARS intervention improved pulmonary artery remodeling and PA smooth muscle layer thickness (Figure 2(g)).

### 3.3. ARS Administration Improved Vascular Relaxation in MCT-PH Rats

**Figure 3:**
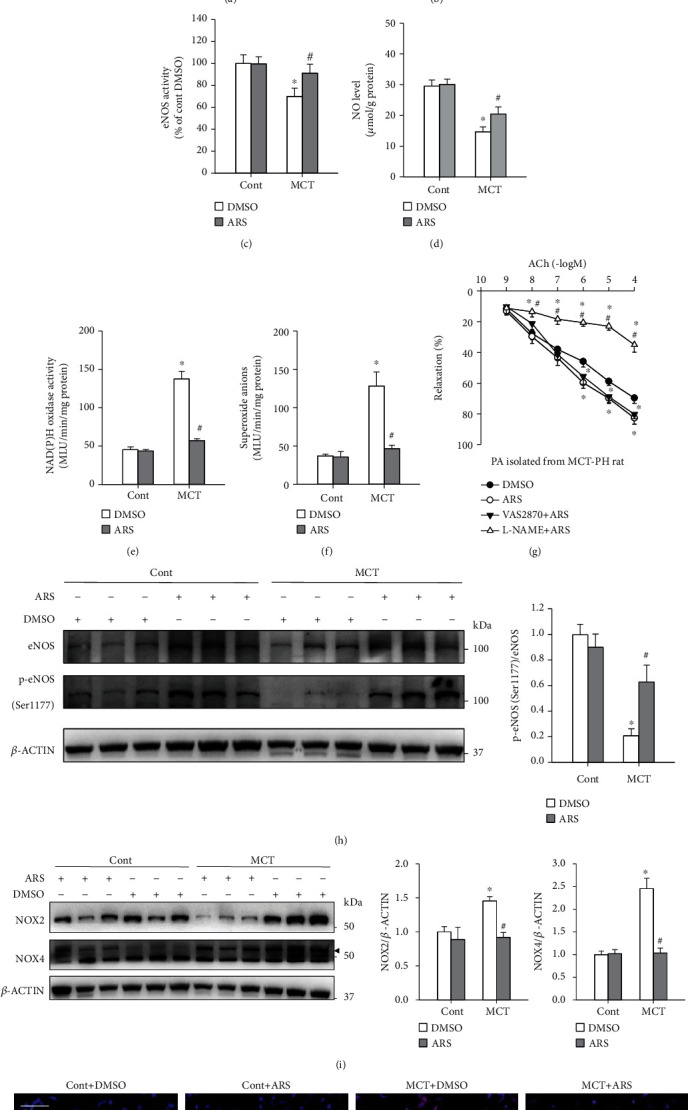
ARS improves the relaxation of PA isolated from MCT-PH rats through promoting eNOS activity and decreasing ROS levels. Effects of ARS (20 *μ*M) acute treatment on PAs (a) basal vascular tension, (b) ACh-induced dose-dependent relaxation after PGF 2*α* (1-5 *μ*M) induced pre-contraction, (c) eNOS activity, (d) NO level, (e) NAD(P)H oxidase activity, and (f) superoxide anion level in control and MCT-pH rats. (g) Effects of pretreatment with VAS2870 (10 *μ*M) and L-NAME (10 *μ*M) on ACh-induced relaxation response to ARS in PAs isolated from MCT-PH rats. Effects of chronic application of ARS (60 mg/kg/day, i.p.) for 4 weeks on eNOS activity (p-eNOS/eNOS) (h), NAD(P)H oxidase subunits NOX2 and NOX4 expressions (i), and ROS levels detected by dihydroethidium (DHE) staining (j) in lung tissues of Cont and MCT-PH rats. Scale bar, 20 *μ*m. Values are mean ± SEM. ^∗^*P* < 0.05 compared with Cont. ^#^*P* < 0.05 compared with DMSO. (Only for (g), ^∗^*P* < 0.05 compared with DMSO. ^#^*P* < 0.05 compared with ARS.) For (a) and (b), *n* = 7 for each group. For (c)–(i), *n* = 6 for each group.

Incubation of PA with ARS (20 *μ*M) for 1 h decreased the basal vascular tension in MCT-induced PH rats significantly (Figure 3(a)). The ACh-induced endothelium-dependent relaxation of PA was attenuated in MCT-PH rats compared with control rats. Treatment with ARS significantly improved ACh-induced dose-dependent relaxations in PA in MCT-PH rats but had no significant effect on control rats (Figure 3(b)). L-NAME pretreatment abrogated the improved effect of ARS on endothelium-dependent relaxation of PA isolated from MCT-PH rats, while VAS2870 failed to enhance this effect (Figure 3(g)).

### 3.4. ARS Administration Preserved eNOS Activity and NO Level but Decreased NAD(P)H Oxidase Activity and ROS Levels in PA Isolated from MCT-PH Rats

Compared with controls, the MCT-PH rats showed declined eNOS activity (Figure 3(c)), p-eNOS/T-eNOS (Figure 3(h)), and NO levels (Figure 3(d)), elevated NAD(P)H oxidase activity (Figure 3(e)), and increased levels of NAD(P)H oxidase subunits (NOX2 and NOX4) expressions (Figure 3(i)) and ROS production (Figures 3(f) and 3(j)) in PAs or lung tissues. Either acute treatment with ARS on PAs for 1 h (Figures 3(c)–3(f)) or chronic ARS administration in rats for 28 days (Figures 3(h)–3(j)) increased or normalized eNOS activity and NO levels. It decreased NAD(P)H oxidase activity and expressions as well as ROS production in PAs of MCT-PH rats (Figures 3(c)–3(f)).

### 3.5. ARS Treatment Attenuated the Development of SuHx-Induced Pulmonary Hypertension

**Figure 4:**
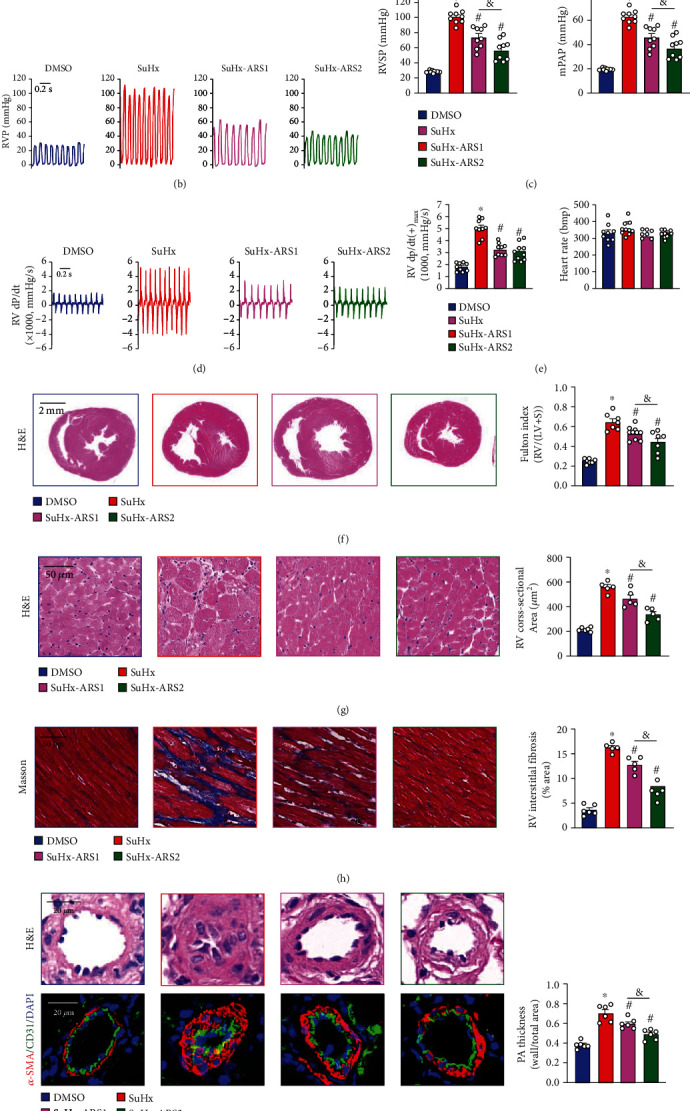
ARS treatment improves hemodynamics and right ventricular hypertrophy in SuHx-PH rat model. (a) Demonstration of experimental grouping and medication duration. Male rats (200 ± 20 g) were subjected to normoxia (Nor) or SuHx (10% O_2_ 3 weeks plus normoxia 6 weeks after subcutaneous injection of Sugen5416 at 20 mg/kg) for 9 weeks. In SuHx-ARS1 group, the application of ARS (60 mg/kg/day) was performed in the 7^th^-9^th^ week. The rats in the SuHx-ARS2 group were treated with ARS in the 4^th^-6^th^ week. (b) Representative tracings showing RVP. (c) Summarized data showing RVSP/mPAP. (d) Representative RV ± dP/dt. (e) Summarized values of RV dp/dt(+)_max_ and heart rate. (f) Representative H&E images showing cardiac morphology changes (scale bar = 2 mm, left panel) and summarized data of Fulton index (right panel). (g) Representative H&E staining images of right ventricular cross-section area (scale bar = 50 *μ*m, left panel) and summarized data showing myocyte cross-sectional area (right panel). For each experiment, we analyzed 5-10 fields (500-1000 cells) of view per rats. (h) Masson's staining images showing collagen deposition (scale bar = 50 *μ*m, left panel) and summarized data showing the percentage of collagen deposition area (right panel). (i) Representative H&E staining and immunofluorescence images of lung sections showing PA remodeling (left panel), summarized data showing media thickness of the small PAs (right panel) in control and SuHx rats receiving DMSO or ARS. Values are mean ± SEM. ^∗^*P* < 0.05, compared with DMSO group; ^#^*P* < 0.05, compared with SuHx group; ^&^*P* < 0.05, SuHx-ARS1 compared with SuHx-ARS2. The numbers of experiments (*n*) for each group are indicated in each bar.

To further validate the effects of ARS on different experimental pulmonary hypertension models, we established Su5416 combined with chronic hypoxia induced PH rat model. We treat SuHx-PH rats with ARS in two ways to observe the therapeutic effects of ARS on PH at later period and early stage, respectively (Figure 4(a)): first, applying ARS in the last 3 weeks under normoxia (SuHx-ARS1) and second, applying ARS for 3 weeks at once after leaving the hypoxic chamber (SuHx-ARS2). We found that the values of RVSP/mPAP (Figure 4(c)) and RV dP/dt(+)_max_ (Figure 4(e)) of SuHx-induced PH rats significantly increased, while the heart rates had no significant change compared to controls. ARS treatment either in SuHx-ARS1 group or SuHx-ARS2 group decreased these indexes (Figures 4(b)–4(e)). ARS treatment in both routes consistently and significantly alleviated the increased right ventricular hypertrophy which was exhibited in heart H&E images and Fulton index [RV/(LV + S)] (Figure 4(f)), as well as RV wall cross-section (Figure 4(g)) in SuHx-PH model. In addition, both ARS invention ways markedly declined RV interstitial fibrosis which was exhibited from the Masson staining section (Figure 4(h)) in SuHx-PH model. As shown in Figure 4(i), SuHx also resulted in severe pulmonary arteriole remodeling, which was indicated by thickening PA media and lumen stenosis compared to the DMSO group, while both ARS interventions significantly inhibited PA remodeling induced by SuHx-PH. It is worth mentioning that the application of ARS at early stage of PH (SuHx-ARS2) is more effective than application of ARS in later period (SuHx-ARS1), because SuHx-ARS2 decreased RVSP/mPAP and inhibited RV and PA remodeling to a greater extent than SuHx-ARS1 (Figure 4).

### 3.6. DHA Administration Ameliorated MCT-Induced Pulmonary Pathophysiology

**Figure 5:**
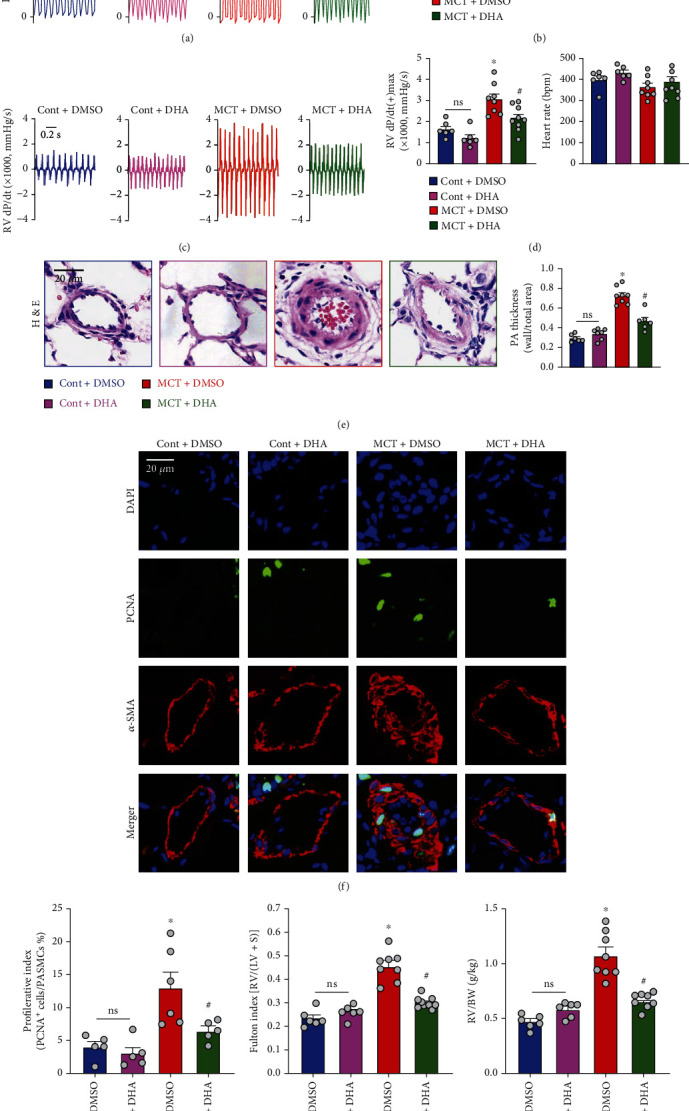
DHA administration ameliorated MCT-induced pulmonary pathophysiology. Male rats (200 ± 20 g) were subcutaneously injected with MCT at a 50 mg/kg single dose. The MCT-treated rats received intraperitoneal administration with DHA (14 mg/kg/day) at the beginning of the experiments. (a) Representative tracings showing RVP. (b) Summarized data showing RVSP/mPAP. (c) Representative RV ± dP/dt. (d) Summarized values of RV dp/dt(+)_max_ and heart rate. (e) Representative images of pulmonary vascular remodeling as detected by H&E staining in the lung tissues (left panel, scale bar, 20 *μ*m) and quantification analysis of pulmonary arterial wall thickness (right panel). (f) Representative immunofluorescence images showing the colocalization of PCNA (shown in green) levels together with *α*-SMA (shown in red) in PAs. Nucleus (blue) was stained with DAPI. Scale bar, 20 *μ*m. (g) Summarized data showing percentage of PCNA-positive VSMCs. (h and i) Summarized data showing Fulton index [RV/(LV + S)] and the ratio of RV to body weight (RV/BW) in Cont and MCT-PH rats receiving DMSO or DHA. Values are mean ± SEM. ^∗^*P* < 0.05, compared with Cont+DMSO. ^#^*P* < 0.05, compared with MCT+DMSO. Ns: no significant difference. The numbers of experiments (*n*) for each group are indicated in each bar.

To exclude the nonspecific action of ARS, we further treated the rats with its derivative, DHA. Similar to ARS, DHA markedly attenuated MCT-induced elevations of RVSP/mPAP (Figure 5(b)), RV dP/dt(+)_max_ (Figure 5(d)), and pulmonary vascular wall thickness (Figure 5(e)) and still had no significant effect on heart rate. Pulmonary smooth muscle cell (PASMC) proliferation is the main factor of pulmonary vascular thickening. Furthermore, to study the role of DHA on PASMCs proliferation, the cell proliferation marker PCNA-positive cells in the medial wall of pulmonary arteries were quantified via immunofluorescence analysis. We observed that there was an increased proportion of PCNA-positive cells in PAs of the MCT-challenged rats compared to the control rats, which was inhibited by DHA intervention (Figures 5(f) and 5(g)). DHA treatment also significantly reduced MCT-induced increase right ventricular hypertrophy index (Figures 5(h) and 5(i)). DHA had no significant effect on control rats (Figure 5). These results demonstrated the beneficial effects of DHA to ameliorate MCT-induced pulmonary pathophysiology.

### 3.7. DHA Treatment Improved Right Ventricular Remodeling and Right Heart Function

**Figure 6:**
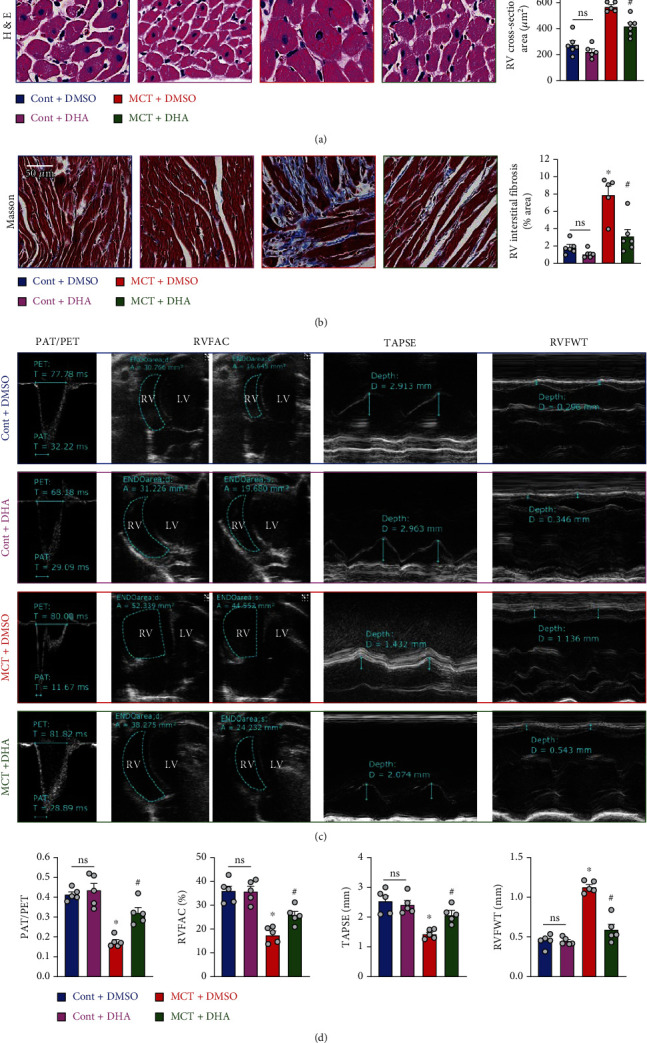
DHA treatment improved right ventricular remodeling and right heart function in MCT-PH rats. (a) Representative H&E staining images of right ventricular sections showing cardiac hypertrophy in rats (left panel, scale bar = 20 *μ*m); summarized data showing the semiquantitative analysis of the RV cross-sectional area (right panel). (b) Representative Masson's staining images of right ventricular sections showing cardiac fibrosis (blue indicates collagen deposition and red represents muscle fibers) in rats (left panel, scale bar = 50 *μ*m); summarized data showing the semiquantitative analysis of the ratio of RV interstitial collagen deposition area (right panel). (c) Representative echocardiographic images (PAT/PET, RVFAC, TAPSE, and RVFWT). (d) Quantification analysis of PAT/PET, RVFAC, TAPSE, and RVFWT in Cont and MCT-PH rats receiving DMSO or DHA. Values are mean ± SEM. ^∗^*P* < 0.05, compared with Cont+DMSO; ^#^*P* < 0.05, compared with MCT+DMSO. Ns: no significant difference. The numbers of experiments (*n*) for each group are indicated in each bar.

To further confirm the effects of DHA on right ventricular remodeling and heart function, we performed cardiac section staining and echocardiographic assessment. We found that MCT-induced RV myocardial hypertrophy (Figure 6(a)) and interstitial fibrosis (Figure 6(b)) were also inhibited by DHA treatment. Echocardiography, an important noninvasive examination method, is usually used for diagnosis of pulmonary hypertension [31, 35]. Before the measurement of hemodynamic parameters, we performed echocardiography on rats to check right heart function. We observed that the ratio of pulmonary acceleration time (PAT) to pulmonary ejection time (PET) (PAT/PET), RV fractional area change (RVFAC), and tricuspid annular plane systolic excursion (TAPSE) was decreased, while right ventricle free wall thickness (RVFWT) was increased in MCT-induced PH rats compared to control rats. DHA administration significantly revised them (Figures 6(c) and 6(d)). These evidences demonstrated that DHA treatment improved the impairment of right cardiac function induced by MCT. Collectively, these results also suggested that DHA could serve as a potential drug through improving the right heart function to prevent PH.

### 3.8. DHA Inhibited hPASMC Proliferation and Migration

**Figure 7:**
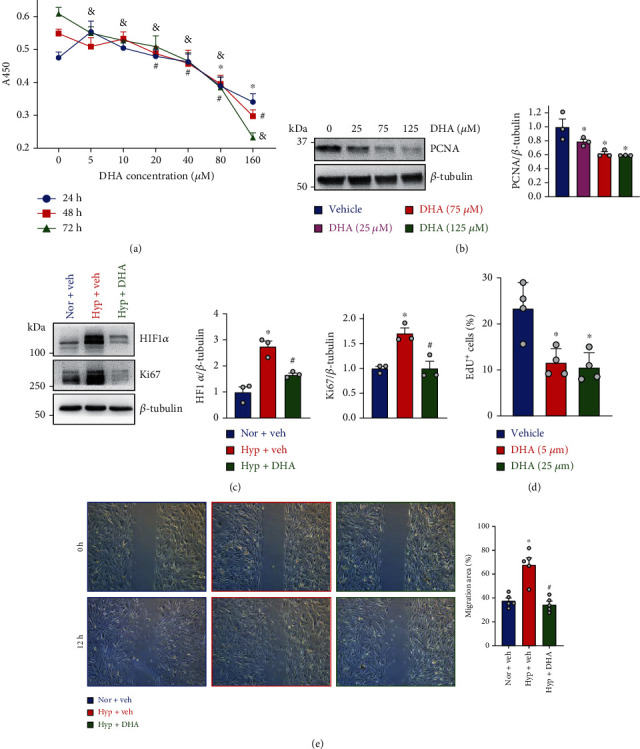
DHA treatment significantly inhibited proliferation and migration of hPASMCs. (a) CCK8 assay showing cell viability of hPASMCs treated with DHA in different concentrations (0, 5, 10, 20, 40, 80, and 160 *μ*M) for 24 h, 48 h, and 72 h under normal condition, respectively. Values are mean ± SEM; ^∗^*P* < 0.05, ^#^*P* < 0.05, and ^&^*P* < 0.05 are compared with DHA (0 *μ*M) in 24 h, 48 h, and 72 h, respectively. *n* = 5 for each group. (b) Representative western blot images and summarized data showing the level of proliferation marker PCNA in hPASMCs treated with DHA in different concentrations (0, 25, 75, and 125 *μ*M) under normal condition. (c) Representative western blot images and statistical data showing the protein expression of Ki67 and HIF1*α* in hPASMCs treated with or without DHA (125 *μ*M) under normoxia (Nor) or hypoxic (Hyp) condition. (d) Summarized data showing the percentage of EdU^+^ cells treated with DHA in different concentrations (0, 5, and 25 *μ*M). (e) Representative images showing hPASMC migration visualized via “wound” or scratch motility assay (left panel). Statistical results showing the migration distance of hPASMC response to DHA (25 *μ*M) under Nor or Hyp. Values are mean ± SEM; ^∗^*P* < 0.05, compared with Nor+Veh or vehicle. ^#^*P* < 0.05, compared with Hyp+Veh. The numbers of experiments (*n*) for each group are indicated in each bar.

Based on the observation that DHA attenuated PASMC proliferation in remodeled small PAs of MCT-PH rats, we then performed *in vitro* experiment to test the effect of DHA on the proliferation and migration of hPASMCs in response to hypoxia. The hPASMCs were first pretreated with different concentrations of DHA for 24 h, 48 h, and 72 h under normal condition, and then, cell viability assay was tested by CCK-8 kit, respectively. As depicted in Figure 7(a), DHA decreased cell proliferation in a concentration-dependent manner. Similarly, further results confirmed that the expression of cell proliferation marker PCNA was also reduced by DHA in a concentration-dependent method (Figure 7(b)). The expression of Ki67 is strongly associated with tumor cell proliferation and growth and is widely used in routine pathological investigation as a proliferation marker. Therefore, hypoxia-induced PASMC proliferation was evaluated by the expression level of Ki67 in this study. Our data showed that the level of Ki67 was increased under hypoxia condition, and DHA pretreatment significantly inhibited hypoxia-induced increase of Ki67 expression (Figure 7(c)). As the main transcription factor of the body in response to hypoxia, HIF*α* activates its downstream target genes through transcription, thereby affecting the occurrence and development of pulmonary hypertension [1, 32]. Meanwhile, DHA treatment also decreased the expression of HIF1*α* of hPASMCs exposed to hypoxia condition (Figure 7(c)). Through flow cytometry analysis, DHA treatment strikingly decreased cell proliferation which was evaluated by the percentage of EdU positive cells (Figure 7(d)). In addition, it was also observed that DHA significantly inhibited the hypoxia-driven hPASMC migration (Figure 7(e)).

## 4. Discussion

In this study, we are the first one who used isolated perfused/ventilated mouse lung to show that ARS is able to attenuate hypoxia or high K^+^ solution-induced pulmonary vasoconstriction in a NO-dependent manner. We also demonstrated that ARS partially reversed MCT or SuHx-induced severe pulmonary hypertension in rats. In addition, we showed that ARS is able to increase eNOS activity and NO levels and decrease NAD(P)H oxidase activity and superoxide anions levels in the pulmonary arteries from MCT-mediated rat model of PH. DHA, the derivative of ARS, has the similar effects to reverse MCT-induced pulmonary hypertension in rats. Therefore, ARS and its derivatives could be used as therapeutic drugs in the treatment of pulmonary hypertension.

Recently, it has been reported that ARS and its derivatives play important protective roles in some cardiovascular diseases apart from their antimalarial activity. ARS or DHA alleviates atherosclerotic lesions in high-fat diet-fed ApoE^−/−^ mice [36, 37], protects against cardiac hypertrophy *in vivo*, blocks angiotensin II-induced cardiac hypertrophy *in vitro* in a concentration-dependent manner [38], and attenuates hepatic steatosis and inflammation in diet-induced obese mice [39, 40]. In this study, we found that the RVSP values and Fulton index (right heart hypertrophy) were increased in both MCT-induced and SuHx-induced PH rats. The sections of RV myocardium with Masson's staining and H&E staining further revealed cardiomyocyte hypertrophy and severe perivascular and myocardial fibrosis in these rat models of PH, suggesting the occurrence of right heart failure. Chronic application of either ARS or DHA decreased the elevated RVSP, RV hypertrophy, and fibrosis in PH. In addition, pulmonary artery media layer in MCT- or SuHx-induced PH significantly proliferated and thickened, resulting in stenosis or even complete occlusion in PA, which signified the occurrence of pulmonary vascular remodeling in PH. DHA or ARS attenuated the pulmonary vascular remodeling in these two rat models of severe PH. All the results indicated that ARS and DHA have the potential therapeutic roles to treat PH.

The SuHx-PH rat model is more closely recapitulates the human pathology than other rodent models [18, 19]. The application of Su5416 combined with hypoxia sets off a cascade of events that leads to structural remodeling of the pulmonary arterioles and progressively worsening hemodynamics and plexiform lesions that are not seen in any other model of PH [23]. In this study, we applied ARS after SuHx at the early stage or at the end-stage of PH to see whether there was any notable difference of the therapeutical effect of ARS. According to the results, we found that, although ARS was beneficial to the treatment of pH overall, application of ARS at the early stage of PH is a little more effective than in a later period.

It has been reported that ARS inhibits the proliferation, migration, and inflammation induced by tumor necrosis factor-alpha (TNF-*α*) in vascular smooth muscle cells (VSMCs) through nuclear factor-kappa B (NF-*κ*B) pathway [41]. ARS alleviates atherosclerotic lesion by the regulation of VSMC phenotype switching [36, 37]. DHA inhibited platelet-derived growth factor-mediated PASMC proliferation and migration in a dose-dependent manner [10]. In this study, we also found that DHA inhibited hypoxia-induced hPASMC proliferation and migration, which further confirmed its therapeutic capability in treating vascular remodeling using our *in vitro* cell culture model.

Both the high K^+^ solution and hypoxia can induce PA contraction and are usually used to evaluate the PA contractile function. In this study, we found that ARS inhibited both high K^+^ solution and hypoxia-induced PA constriction, which indicated the potential beneficial effect of ARS to inhibit the sustained vasoconstriction of PA in PH. In addition, studies have reported that ARS and its derivatives play protected roles in endothelial cells (ECs). DHA has the antihypoxic effect on pulmonary artery endothelial cells [11]; artesunate has a protective effect on LPS induced human umbilical vein endothelial cells injury [42] and also remarkably inhibits the proliferation and differentiation of ECs in a dose-dependent form [43]. ACh stimulates ECs to release vasodilating factors such as NO and then induce VSMCs relaxation, which is usually used to evaluate vascular endothelial function [44–46]. Here, we found that compared with control rats, ACh-induced endothelium-dependent relaxations in PA of MCT-PH rats were significantly attenuated, suggesting the occurrence of endothelial dysfunction in PH. ARS pretreatment on PAs significantly improved the endothelium-dependent relaxations in PH, suggesting that ARS had the ability to restore the endothelial function in pulmonary hypertension. Furthermore, ARS decreased the basal vascular tension and facilitated the relaxation of PA, which might be the consequences of improved endothelial function by ARS.

The mechanisms of the restorative effects of ARS or DHA on ECs and VSMCs have not been determined yet. It has been reported that artesunate, another derivative of ARS, mitigated hypoxia-/reoxygenation-mediated increase in ROS levels in alveolar macrophages [16], and treatment of hemorrhagic shock rats with artesunate enhanced the phosphorylation of eNOS [17]. In this study, we found that the NAD(P)H oxidase activity and ROS levels of PAs in MCT-induced PH rats were higher, while the eNOS activity and NO production were significantly lower than that in control rats. Both acute treatment with ARS on PAs for 1 h or chronic ARS administration in rats for 28 days increased or normalized eNOS activity and NO levels and decreased NAD(P)H oxidase activity and expression levels as well as ROS levels in PAs of MCT-PH rats. Furthermore, L-NAME, a NOS inhibitor, nearly abrogated the effects of ARS on PA contraction and relaxation, which indicated the effects of ARS were NO dependency. We also found that the NAD(P)H oxidase inhibitor, VAS2870, failed to enhance the effects of ARS on PA contraction and relaxation. In addition, VAS2870 and ARS might have the same ability to inhibit the NAD(P)H oxidase, and VAS2870 cannot further enhance the effects of ARS. From these findings, we deduced that ARS might improve endothelial function, facilitate the relaxation, prevent vasoconstriction of PA in PH through inhibiting NAD(P)H oxidase derived ROS production, and activate the eNOS to release NO.

There are several limitations to this study. First, we have not measured eNOS and NAD(P)H activity in SuHx-mediated pulmonary hypertension in rats. Second, it is still unknown about how ARS affects eNOS and NAD(P)H activity or generation of ROS. Thus, the precise molecule mechanisms in activating eNOS and inhibiting ROS production of ARS may warrant our further studies.

In conclusion, we demonstrated that ARS or DHA can partially reverse severe experimental pulmonary hypertension in rats via regulation of pulmonary vasoconstriction and vasodilatation as well as pulmonary vascular remodeling through eNOS-NO and NAD(P)H-ROS signaling pathways.

## Data Availability

The data that support the findings of this study are available from the corresponding author upon reasonable request.
